# Phytosterols and inulin-enriched soymilk increases glucagon-like peptide-1 secretion in healthy men: double-blind randomized controlled trial, subgroup study

**DOI:** 10.1186/s13104-018-3958-5

**Published:** 2018-11-29

**Authors:** Noppadol Kietsiriroje, Krisana Kanjanahirun, Jirateep Kwankaew, Ratikorn Ponrak, Supamai Soonthornpun

**Affiliations:** 1 Division of Endocrinology and Metabolism, Department of Internal Medicine, Faculty of Medicine, Prince of Songkla University, Hat Yai, Songkhla, 90110 Thailand; 2 Internal Medicine Clinic, Samitivej Srinakarin Hospital, Bangkok, 10250 Thailand; 3 Internal Medicine Clinic, Maharaj Nakhon Si Thammarat Hospital, Nakhon Si Thammarat, 80000 Thailand

**Keywords:** Phytosterols, Inulin, Glucose metabolism, Glucagon-like peptide-1 secretion, Insulin secretion

## Abstract

**Objective:**

The study aimed to determine the effect of phytosterols and inulin on plasma glucose, insulin, and GLP-1 levels among healthy men after consuming phytosterols and inulin-enriched soymilk for 8 weeks.

**Results:**

A total of 26 men at least 20 years old were randomly assigned into the 2 g/day of phytosterols and 10 g/day of inulin-enriched soymilk (intervention) group or into the standard soymilk (control) group. In the intervention group, the area under the curve of Glucagon-like peptide-1 secretion increased significantly, compared to its baseline (p = 0.003). The area under the curve of insulin secretion also increased but it did not meet statistical significance (p = 0.118). The area under the curves of plasma glucose were similar between pre- and post-test (p = 0.348). In the control group, none of the primary results significantly changed compared to their baseline levels.

*Trial registration* Thai Clinical Trial Registry: TCTR20160319001 date: March 19, 2016, retrospectively registered.

**Electronic supplementary material:**

The online version of this article (10.1186/s13104-018-3958-5) contains supplementary material, which is available to authorized users.

## Introduction

Glucagon-like peptide-1 (GLP-1) is a gut-derived peptide secreted from intestinal L-cells after a meal. The main stimuli of GLP-1 secretion are fat and carbohydrates. Amino acids, bile acids, and short-chain fatty acids (SCFAs) produced by bacterial fermentation of dietary fiber are also able to stimulate GLP-1 secretion through different specific receptors and pathways [1]. Inulin is a soluble, indigestible fiber which can be fermented by intestinal bacteria. SCFAs are the by-products from this process.

The effects of indigestible fibers such as inulin on GLP-1 secretion in the human model have not been well-elucidated. Only a few small studies attempted to demonstrate the effect of inulin on GLP-1 secretion in different samples of healthy participants [2, 3], overweight or obese participants [4], and pre-diabetes participants [5].

Phytosterols are sterols from plants that are structurally related to cholesterol. They competitively inhibit cholesterol absorption in the intestinal lumen leading to a decrease in the plasma cholesterol level [6]. To date, the benefit of phytosterols is limited mainly to cholesterol reduction. No research has mentioned the impact of phytosterols consumption on GLP-1 or insulin secretion.

Soymilk is popular and widely available among Asian countries. Therefore, it is a suitable food carrier of nutrients such as phytosterols and inulin to increase metabolic values of soymilk products. This subgroup study aims to determine the effect of phytosterols and inulin on plasma glucose, insulin, and GLP-1 levels among healthy men.

## Main text

### Materials and methods

#### Study design

This study is a sub-study of *Effect of phytosterols and inulin*-*enriched soymilk on LDL*-*cholesterol in Thai subjects: a double*-*blinded randomized controlled trial* which enrolled 240 adult volunteers from the outpatient department and the Endocrinology and Metabolism Unit of Songklanagarind Hospital at Prince of Songkla University from 28 May 2013 to 5 June 2014. The randomization was performed by a third party and simple random sampling was used to generate a number sequence by the sponsor with an allocation ratio of 1:1. In the main study, 120 participants (101 women and 19 men) were allocated into the intervention group which received 2 g/day of phytosterols and 10 g/day of inulin-enriched soymilk. The other 120 participants (103 women and 17 men) were allocated into the control group which received standard soymilk [7].

Only men were eligible for the current study, to avoid the impact of menstruation cycle on insulin resistance [8]. All 36 men were given the informed consent form. Fourteen out of 19 participants in the intervention arm and 12 out of 17 participants in the control arm provided written informed consents. Details of the enrollment of the participants are shown in the additional file (see Additional file 1).

All participants were at least 20 years old and nondiabetic. The details of the exclusion criteria and soymilk products are described in the additional file (see Additional file 1). Neither specific dietary control nor exercise intervention was given in this study. All participants could maintain their usual habits while they attended the study.

The oral glucose tolerance test using 75 g of glucose (75 OGTT) is the standard beta-cell function assessment [9]. The 75 OGTT was conducted in all participants at the time of randomization (week 0) and at the end of the study (week 8). Blood sampling for biochemistry tests, that included plasma glucose, insulin, and GLP-1 levels, were drawn after a 12-h fasting period and at 30, 60, 90, and 120 min after administration of the 75 OGTT.

The primary outcomes are the changes in the areas under the curves (AUCs) of plasma glucose, insulin, and GLP-1 before and after the consumption of the phytosterols and inulin-enriched soymilk for 8 weeks. The secondary outcomes are the differences in the AUC of plasma glucose, insulin, and GLP-1 between the intervention and control groups.

#### Biochemical testing

Venous blood samples were drawn at baseline after a 12-h fast at week 0 and week 8. After baseline venous blood samples were collected, the 75 OGTT was done and venous blood samples were sequentially collected at 30, 60, 90, and 120 min. Serum aliquots for fasting plasma glucose (FPG) were stored at room temperature and transported to the laboratory for testing on the same day. They were measured by enzymatic in vitro assay for direct determination and the intra-assay coefficients of variation (CV) ranged from 1.48 to 1.52%.

Serum aliquots for insulin and GLP-1 were stored at − 80 °C until all venous blood samples from all participants were completely collected. Measurements of insulin and GLP-1 levels were done simultaneously from the same serum aliquot. The insulin levels were measured by in-house radioimmunoassay and the intra-assay CV ranged from 0.8 to 1.5%. The GLP-1 levels were measured by enzyme-linked immunosorbent assay (Multi Species GLP-1 Total ELISA, EZGLP1T-36 K, Merck) using a Multiskan GO reader. The intra-assay CV of the GLP-1 ranged from 7.49 to 10.72%.

Homeostasis model assessment-B (HOMA-B) and homeostasis model assessment of insulin resistance (HOMA-IR) were calculated using standard formulas. The HOMA-IR value used 1.56 as the cut-off value to determine insulin resistance in Thai men [10].

#### Statistical analysis

Descriptive data are shown as mean, median, standard deviation (SD), and interquartile range (IQR). The Chi square test or Fisher’s exact test, and independent t test or Mann–Whitney U test were used to identify differences between the baseline characteristics which were categorical and continuous data, respectively. The AUC of each variable was calculated by the trapezoid time 0–t_max_ method. The differences of the AUCs between the study groups and between pre- and post-intervention within the groups were determined by the mixed effects linear regression model. The mixed effects linear regression model was also used to determine the differences in mean scores of the variables, between the pre- and post-test, at each time-point. A p value < 0.05 indicated statistical significance. R-3.4.1 for Windows and STATA version 14.0 were used for the data analyses.

### Results

Twenty-six male volunteers were screened and enrolled into the study (14 participants in the intervention group and 12 participants in the placebo group). All participants completed the 8-week follow-up and were included into the primary analysis; however, one participant with an extreme fasting insulin value in the placebo group was removed before the primary analysis was performed. The participant with the extreme value of fasting insulin was detected by boxplot test. A re-analysis of the primary outcomes, which included the extreme value participant, was also performed to confirm that this exclusion did not change the primary results (see Additional file 2].

The baseline characteristics of the participants in each group are summarized in Table 1. There were no differences in the baseline characteristics between the groups except waist circumference which was higher in the control group (p = 0.028).

**Table 1:** Comparisons of baseline characteristics between groups (N = 26)

Baseline characteristics	Mean ± SD, median [IQR], n (%)		p-value
	Intervention (n = 14)	Control (n = 12)	
Age, year	43.14 ± 9.80	43.00 ± 13.20	0.98
Waist circumference, cm	87.36 ± 6.26	94.33 ± 8.93	0.03^*^
BMI, kg/m^2^	24.84 ± 2.60	27.21 ± 3.42	0.06
HTN, n (%)	1 (7.14%)	1 (8.33%)	1.00
DLP, n (%)	1 (7.14%)	2 (16.67%)	0.58
SBP, mmHg	120.50 [115.75, 125.25]	127.5 [119.25, 142.00]	0.09
DBP, mmHg	78.07 ± 12.58	82.67 ± 12.51	0.36
TC, mg/dL	237.36 ± 33.28	255.17 ± 32.47	0.18
TG, mg/dL	121.00 [89.25, 168.25]	178.00 [102.75, 244.75]	0.21
HDL-c, mg/dL	52.93 ± 11.65	52.00 ± 12.42	0.85
LDL-c, mg/dL	173.50 ± 28.31	181.00 ± 33.37	0.54
AST, U/L	21.50 [19.00, 29.50]	22.50 [18.00, 30.25]	0.71
ALT, U/L	29.00 [19.00, 33.25]	40.5 [20.00, 51.75]	0.21
FPG, mg/dL	92.71 ± 8.21	89.92 ± 5.30	0.32
fasting insulin, mIU/L ^†^	6.75 ± 2.43	8.27 ± 4.62	0.30
fasting GLP-1, pg/mL ^†^	43.50 ± 19.51	50.19 ± 21.90	0.48
HOMA-B ^†^	74.47 [58.85, 110.15]	77.57 [60.23, 146.16]	0.70
HOMA-IR ^†^	1.47 [1.00, 2.11]	1.68 [1.00, 1.95]	0.36
Insulin resistance, n (%) ^†^	7 (50.00%)	7 (63.63%)	0.70

^*^ Independent t-test, p < 0.05

^†^One extreme value participant in the placebo group was removed (n = 11)

**Fig. 1:**
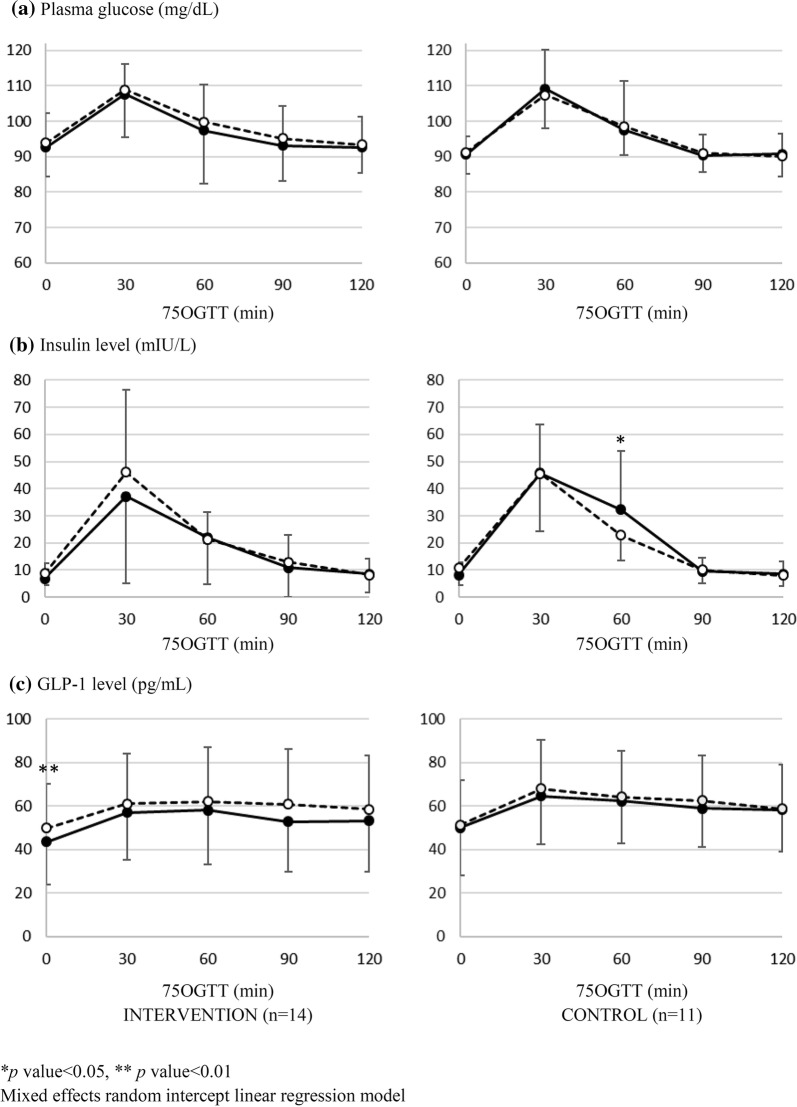
Mean scores from the 75 OGTT of: **a** plasma glucose, **b** insulin, and **c** GLP-1 levels, before and after the soymilk consumption; Intervention (n = 14), Control (n = 11); *Black circle* (●) represents the pre-test values (week 0); *white circle* (○) represents the post-test values (week 8)

Only the mean score of basal GLP-1 level in the intervention group was significantly higher than the pre-test value (43.50 ± 19.51 vs. 49.90 ± 20.03 pg/mL, p = 0.002) (Fig. 1). The full analysis comparing the mean scores of plasma glucose, insulin, and GLP-1 levels, between the pre- and post-tests, at each time-point, was reported in more detail (see Additional file 3).

**Fig. 2:**
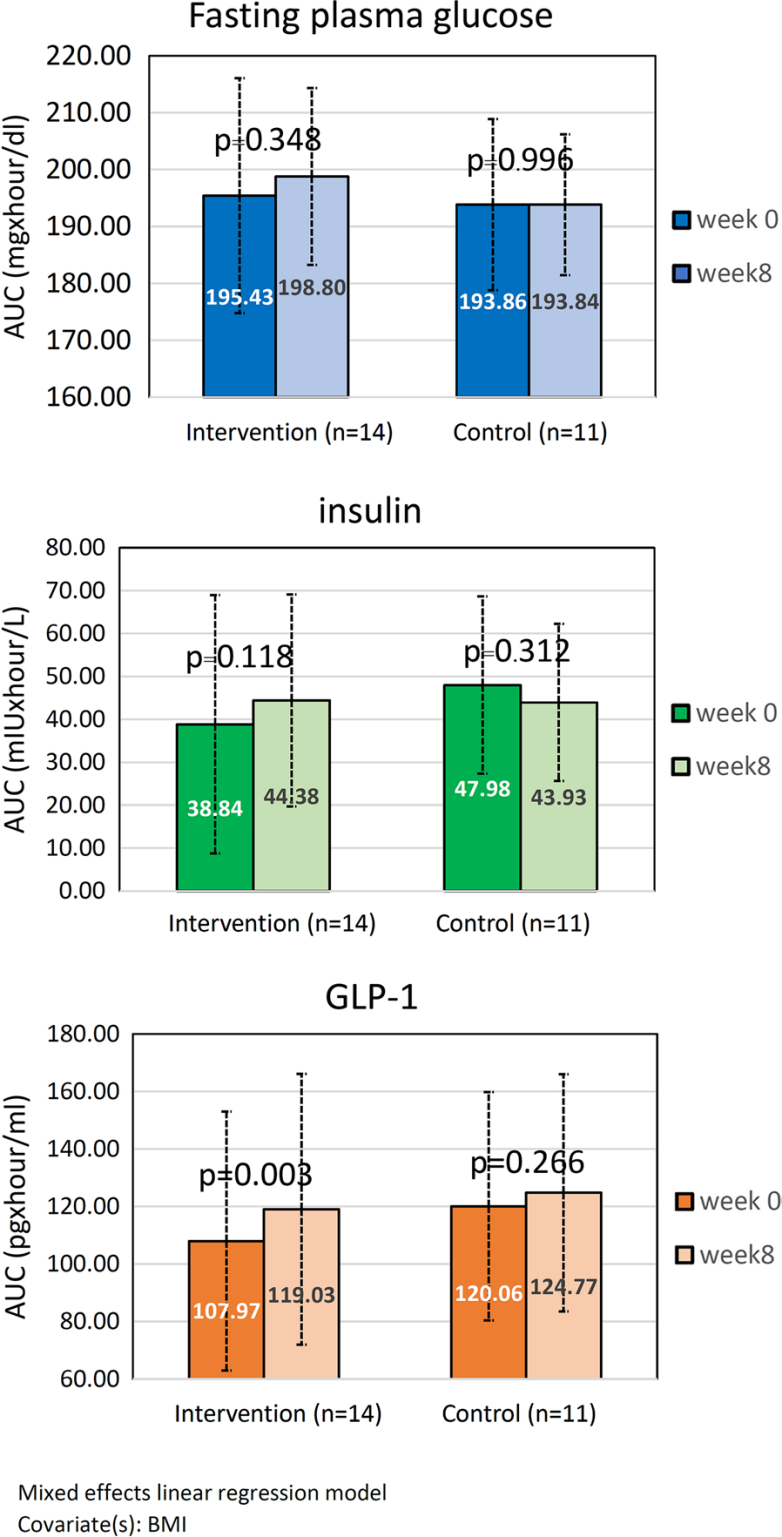
Area under the curve (time _0–2 h_) of plasma glucose, insulin, and GLP-1 at week 0 and week 8 between the intervention group and control group (n = 25)

The baseline AUCs of plasma glucose, insulin, and GLP-1 were not different between the groups (p = 0.835, 0.400, and 0.490, respectively). In the intervention group, the AUC of the GLP-1 increased significantly compared to the baseline (107.97 ± 45.00 vs. 119.03 ± 47.13 pg × h/mL, p = 0.003). Although the AUC of insulin also increased, it did not reach statistical significance (38.84 ± 30.10 vs. 44.38 ± 24.69 mIU × h/L, p = 0.118). The AUCs of plasma glucose were similar between the pre- and post-tests (195.43 ± 20.67 vs. 198.80 ± 15.36 mg × h/dL, p = 0.348). In the control group, the AUCs of GLP-1, insulin, and plasma glucose did not change significantly compared to their baseline levels (Fig. 2).

The mixed effects linear regression model was used to adjust the effects of waist circumference and BMI on the primary outcomes. From the model, neither BMI nor waist circumference had a substantial effect on the primary results.

The mean differences of the AUCs of plasma glucose, insulin, and GLP-1 before and after the soymilk consumption were not significantly different between the groups (Additional file 4).

### Discussion

The study showed that the AUC of the GLP-1 level increased significantly after ingestion of the phytosterols and inulin-enriched soymilk for 8 weeks; however, the insulin and plasma glucose levels did not significantly increase from their baselines.

Most of the previous research that studied the effects of phytosterols on glucose and insulin levels came from animal or in vitro studies. Oral administration of ɣ-sitosterol or β-sitosterol in streptozotocin-induced diabetic rats could significantly decrease blood glucose and glycosylated hemoglobin levels and increase plasma insulin levels [11, 12]. Although some in vitro studies tried to propose cellular mechanisms of phytosterols on glucose metabolism by AMPK activation and PPAR-gamma transcription leading to enhanced insulin sensitivity, the beneficial effect of phytosterols on glucose metabolism is still unclear [13, 14].

While the mechanism of phytosterols on GLP-1 secretion is unclear, inulin-type fructans have more promising evidence in both animal and human studies. Short-chain fatty acids, derived from inulin fermentation by gut microbiomes, promoted proglucagon gene expression in mature intestinal L-cells and promoted L-cell differentiation in the proximal colon of mice [15].

Consumption of inulin or oligofructose also increased the colonic SCFAs in human participants and caused an increment of GLP-1 concentration [2, 4]. The current study also showed that the elevation of GLP-1 secretion was noticeable from the basal state and persisted during the entire 75 OGTT. Therefore, we believe that the consumption of inulin increased proglucagon gene expression in human intestinal L-cells as it did in the animal study.

Interestingly, the increment of GLP-1 secretion by the phytosterols and inulin-enriched soymilk consumption did not have beneficial effects on either insulin secretion or plasma glucose reduction. It may be explained by the magnitude of GLP-1 increment was possibly too minimal to demonstrate any glucose lowering effect in healthy, nondiabetic participants. Other studies, that included either overweight/obese or prediabetic participants, also showed that the consumption of oligofructose or inulin could not demonstrate a glucose lowering effect [4, 5]. The current study also included both overweight and prediabetic participants; therefore, the glucose outcomes were consistent among these studies.

While the neutral impact on the glucose lowering effect was consistent, the impact of oligofructose or inulin on insulin secretion, however, was different among these studies. In one study, the consumption of a standard meal containing 30 g/day of inulin significantly increased both GLP-1 and insulin levels among prediabetic participants [5], whereas another study showed that the consumption of a standard meal containing 30 g/day of oligofructose did not significantly increase either the GLP-1 level or the insulin level [4]. The current study demonstrated that soymilk containing 2 g/day of phytosterols and 10 g/day of inulin significantly increased only the GLP-1 level but did not significantly increase the insulin level. The different outcomes among studies were possibly influenced by the differences of food carriers, amounts of inulin or the types of inulin.

## Limitations

The current study has some limitations. The sample size was small; however, the pre-post study design had adequate power to detect a change in the AUC of GLP-1 within the groups. The soymilk product was combined with both phytosterols and inulin, the interaction between both active ingredients on GLP-1 secretion could not be determined. The subgroup study included only male participants. Since neither dietary control nor exercise intervention was given in this study, the influence of different habits on the primary outcomes is unknown. The participants in the control group had higher values of waist circumference and BMI; however, the changes of GLP-1 or insulin were not influenced by either waist circumference or BMI. The precision of the GLP-1 assay was quite low since the intra-assay CV was over 10% (10.72%) at the low reference level. This study did not measure the amount of fecal SCFAs, thus the mechanism of colonic SCFA derived by inulin fermentation that causes an increment of GLP-1 concentration in human participants, could not be proved by this study.

## Additional files

**Additional file 1.** Participant enrollment, exclusion criteria, and soymilk products’ information.

**Additional file 2.** The Analyses of primary outcomes before and after exclusion of the extreme value in the control group.

**Additional file 3.** Comparison of mean scores (A), or mean differences (B) of variables between pre- and post-test values at each time-point within group (n = 25).

**Additional file 4.** Mean differences of AUC of plasma glucose, insulin and GLP-1 between both groups (n = 25).

## Abbreviations

GLP-1: glucagon-like peptide-1
AUC: area under the curve
75 OGTT: 75-gram oral glucose tolerance test
SCFA: short-chain fatty acids
FPG: fasting plasma glucose
CV: coefficients of variation
HOMA-B: homeostasis model assessment-B
HOMA-IR: homeostasis model assessment of insulin resistance
SD: standard deviation
IQR: inter-quartile range
BMI: body mass index
HTN: hypertension
DLP: dyslipidemia
SBP: systolic blood pressure
DBP: diastolic blood pressure
TC: total cholesterol
TG: triglyceride
HDL-c: high-density lipoprotein cholesterol
LDL-c: low-density lipoprotein cholesterol
AST: aspartate transaminase
ALT: alanine transaminase
