# Supplementary material for: Phytosterols and inulin-enriched soymilk increases glucagon-like peptide-1 secretion in healthy men: double-blind randomized controlled trial, subgroup study
Source: BMC Res Notes. 2018 Nov 29;11:844. doi: 10.1186/s13104-018-3958-5 (PMC6267084; doi:10.1186/s13104-018-3958-5)
Supplement: Supplementary file 1 — Additional file 1. Participant enrollment, exclusion criteria, and soymilk products’ information. [file 13104_2018_3958_MOESM1_ESM.docx]

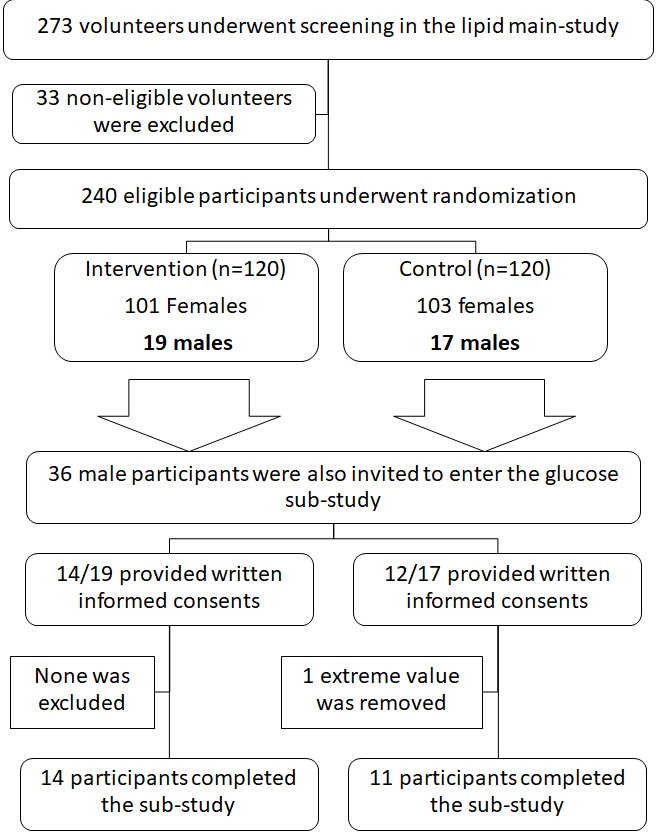


**Fig. S1**. Enrollment of the participants (N = 26)

**Exclusion criteria**

The exclusion criteria were those subjects who had: 1) established cardiovascular disease, 2) any type of diabetes mellitus, 3) gastrointestinal dysmotility, 4) abnormal gastrointestinal digestion or absorption, 5) an allergy to soymilk and 6) secondary hyperlipidemia such as hypothyroidism, nephrotic syndrome or hepatic disease. The withdrawal or termination criteria are subjects who: 1) intended to withdraw, 2) lost to follow-up, 3) had elevated transaminase enzymes >3 fold of the upper normal limits, 4) had reduced glomerular filtration rate ≥1 stage by the Kidney Disease Outcomes Quality Initiative criteria, 5) were unable to tolerate adverse events from soymilk products and 6) had compliance less than 80 % per visit.

**Soymilk products**

The phytosterols and inulin-enriched soymilk (UHT SOY MILK WITH PHYTOSTEROL; DNA®, Thai FDA food registration number 60-1-05841-2-0089) and standard soymilk in this study were supplied by Dairy Plus Company Limited. A serving unit of both soymilk products contained 180 ml of soymilk, 4.5 g of fat, 5 g of protein and 12 g of carbohydrate. In addition, a serving unit of the phytosterols and inulin-enriched soymilk contained 1 g of phytosterols and 5 g of inulin. The components of the soymilk products are shown in Tables below

**Table S1.** Macronutrient composition of both soymilk products

Table S2. Sterol composition of UHT SOY MILK WITH PHYTOSTEROL; DNA®

Both soymilk products were concealed at the manufacturing process. The details of the soymilk products were provided below. All subjects were given instructions to drink soymilk products twice daily, once in the morning and once in the evening. The compliance was assessed by counting the used and unused products. The subjects who had less than 80% completeness were determined as noncompliance and would be excluded from the primary analysis.
