# Supplementary material for: Phytosterols and inulin-enriched soymilk increases glucagon-like peptide-1 secretion in healthy men: double-blind randomized controlled trial, subgroup study
Source: BMC Res Notes. 2018 Nov 29;11:844. doi: 10.1186/s13104-018-3958-5 (PMC6267084; doi:10.1186/s13104-018-3958-5)
Supplement: Supplementary file 2 — Additional file 2. The Analyses of primary outcomes before and after exclusion of the extreme value in the control group. [file 13104_2018_3958_MOESM2_ESM.docx]

[A]

[C]

[B]


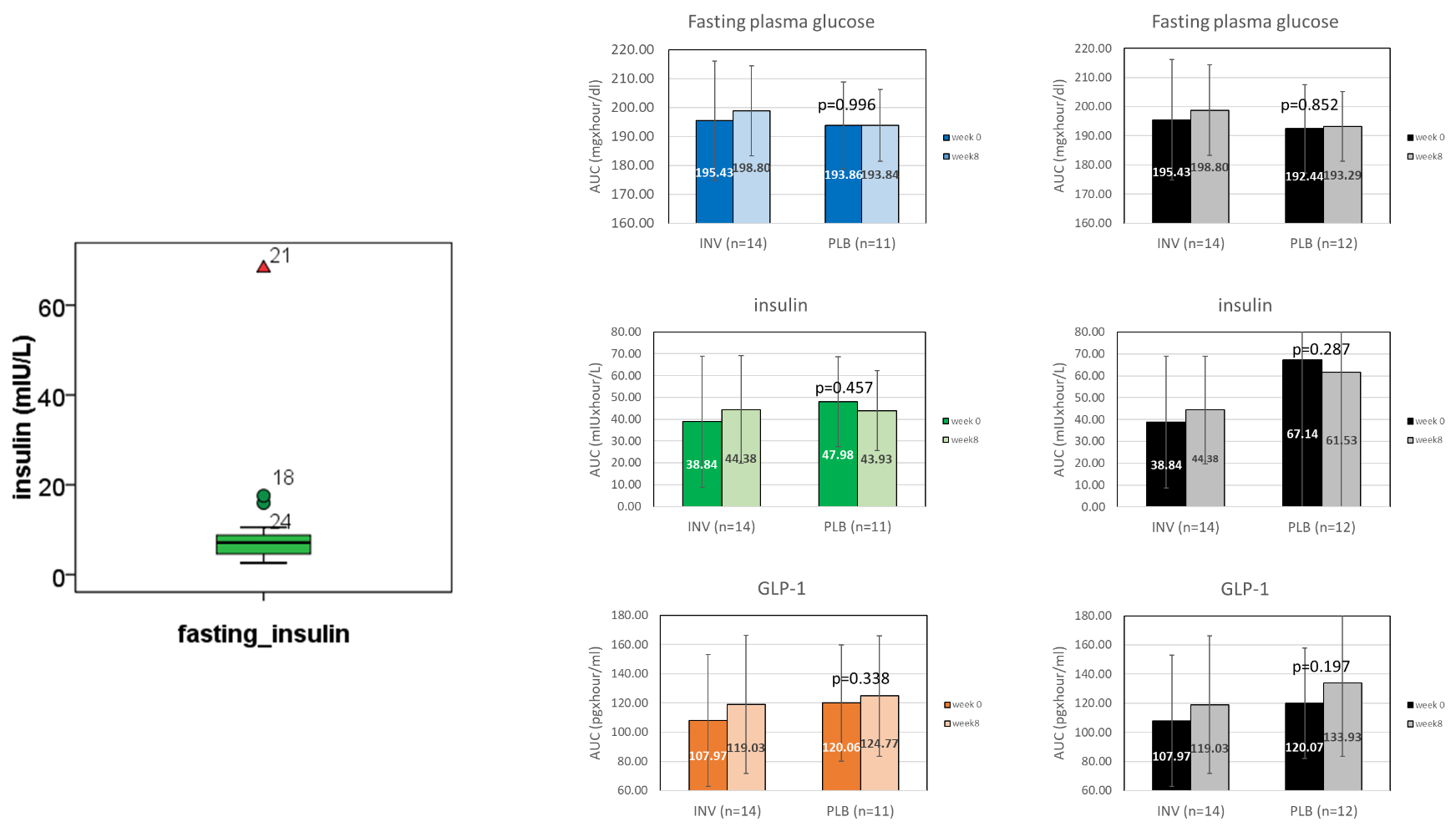


**Additional Fig. S**2 Subject number 21 had an extreme value from the boxplot test and was excluded from the primary analysis (panel A). The primary outcomes after subject number 21 was excluded are shown in panel B. A reanalysis of the primary outcomes which included subject number 21 in the analysis did not change the statistical significance of the pre-post values, fasting plasma glucose, insulin level or GLP-1 in the control group (panel C). *The paired t-test was used to determine the differences of the pre-post values.*
