# Supplementary material for: Phytosterols and inulin-enriched soymilk increases glucagon-like peptide-1 secretion in healthy men: double-blind randomized controlled trial, subgroup study
Source: BMC Res Notes. 2018 Nov 29;11:844. doi: 10.1186/s13104-018-3958-5 (PMC6267084; doi:10.1186/s13104-018-3958-5)
Supplement: Supplementary file 3 — Additional file 3. Comparison of mean scores (A), or mean differences (B) of variables between pre- and post-test values at each time-point within group (n = 25). [file 13104_2018_3958_MOESM3_ESM.docx]

**Additional file S3.** Comparison of: mean scores (A), or mean differences (B) of variables, between pre- and post-test values, at each time-point, within the groups (n = 25).

1. Data demonstrate in mean scores

| time | Intervention (n = 14) | | | | | Control (n = 11) | | | | |
| --- | --- | --- | --- | --- | --- | --- | --- | --- | --- | --- |
|  | Pre- (Week 0) | | Post- (Week 8) | | p value^‡^ | Pre- (Week 0) | | Post- (Week 8) | | p value^‡^ |
|  | Mean | SD | Mean | SD |  | Mean | SD | Mean | SD |  |
| **Plasma glucose (mg/dL)** |  |  |  |  |  |  |  |  |  |  |
| 0 min | 92.71 | 8.21 | 93.93 | 8.40 | 0.555 | 90.55 | 5.07 | 91.27 | 6.05 | 0.794 |
| 30 min | 107.57 | 11.96 | 108.86 | 7.23 | 0.980 | 109.18 | 11.10 | 107.36 | 9.27 | 0.518 |
| 60 min | 97.5 | 15.16 | 99.86 | 10.53 | 0.695 | 97.55 | 13.70 | 98.64 | 8.12 | 0.926 |
| 90 min | 93.07 | 9.96 | 95.21 | 9.01 | 0.750 | 90.36 | 5.89 | 91.00 | 5.39 | 0.982 |
| 120 min | 92.71 | 7.37 | 93.43 | 7.84 | 0.864 | 90.73 | 5.92 | 90.09 | 5.80 | 0.729 |
| **Insulin (mU/L)** |  |  |  |  |  |  |  |  |  |  |
| 0 min | 6.75 | 2.43 | 8.68 | 3.65 | 0.671 | 8.27 | 4.62 | 10.70 | 6.20 | 0.559 |
| 30 min | 37.24 | 31.97 | 45.96 | 30.33 | 0.290 | 45.82 | 17.79 | 45.52 | 21.14 | 0.643 |
| 60 min | 21.90 | 17.16 | 21.33 | 9.98 | 0.696 | 32.18 | 21.72 | 22.84 | 9.28 | 0.046* |
| 90 min | 10.86 | 10.69 | 13.01 | 9.94 | 0.972 | 9.59 | 5.06 | 10.14 | 4.92 | 0.750 |
| 120 min | 8.62 | 6.77 | 8.24 | 6.05 | 0.718 | 8.47 | 4.72 | 8.02 | 3.39 | 0.624 |
| **GLP-1 (pg/mL)** |  |  |  |  |  |  |  |  |  |  |
| 0 min | 43.50 | 19.51 | 49.90 | 20.03 | 0.002** | 50.19 | 21.90 | 51.26 | 20.48 | 0.681 |
| 30 min | 56.90 | 21.89 | 61.00 | 23.00 | 0.426 | 64.69 | 22.32 | 67.92 | 22.50 | 0.558 |
| 60 min | 57.92 | 25.01 | 61.96 | 24.94 | 0.415 | 62.26 | 19.44 | 64.12 | 21.07 | 0.831 |
| 90 min | 52.76 | 22.77 | 60.85 | 25.09 | 0.561 | 58.90 | 17.86 | 62.53 | 20.93 | 0.488 |
| 120 min | 53.23 | 23.33 | 58.59 | 24.77 | 0.720 | 58.35 | 19.23 | 58.69 | 20.18 | 0.841 |
| ‡Mixed effects random intercept linear regression model, neither BMI nor waist circumference had any substantial effect on the outcomes. (**p < 0.01, *p < 0.05) | | | | | | | | | | |

1. Data demonstrate in mean differences

|  | Intervention (n = 14) | | |  | Control (n = 11) | | |
| --- | --- | --- | --- | --- | --- | --- | --- |
| variables | ∆ pre-post | | p value^‡^ | Variables | ∆ pre-post | | p value^‡^ |
|  | Coef. | SE |  |  | Coef. | SE |  |
| Glucose (mg/dL) |  |  |  | Glucose (mg/dL) |  |  |  |
| 0 min | 1.214 | 2.058 | 0.555 | 0 min | 0.727 | 2.781 | 0.794 |
| 30 min | 0.071 | 2.911 | 0.980 | 30 min | -2.545 | 3.933 | 0.518 |
| 60 min | 1.143 | 2.911 | 0.695 | 60 min | 0.364 | 3.933 | 0.926 |
| 90 min | 0.929 | 2.911 | 0.750 | 90 min | -0.091 | 3.933 | 0.982 |
| 120 min | -0.500 | 2.911 | 0.864 | 120 min | -1.36 | 3.933 | 0.729 |
| Insulin (mU/L) |  |  |  | Insulin (mU/L) |  |  |  |
| 0 min | 1.929 | 4.536 | 0.671 | 0 min | 2.435 | 4.164 | 0.559 |
| 30 min | 6.788 | 6.415 | 0.290 | 30 min | -2.727 | 5.889 | 0.643 |
| 60 min | -2.503 | 6.415 | 0.696 | 60 min | -11.771 | 5.889 | 0.046* |
| 90 min | 0.222 | 6.415 | 0.972 | 90 min | -1.879 | 5.889 | 0.750 |
| 120 min | -2.313 | 6.415 | 0.718 | 120 min | -2.883 | 5.889 | 0.624 |
| GLP-1 (pg/mL) |  |  |  | GLP-1 (pg/mL) |  |  |  |
| 0 min | 6.404 | 2.048 | 0.002** | 0 min | 1.073 | 2.611 | 0.681 |
| 30 min | -2.307 | 2.896 | 0.426 | 30 min | 2.164 | 3.692 | 0.558 |
| 60 min | -2.361 | 2.896 | 0.415 | 60 min | 0.786 | 3.692 | 0.831 |
| 90 min | 1.686 | 2.896 | 0.561 | 90 min | 2.559 | 3.692 | 0.488 |
| 120 min | -1.039 | 2.896 | 0.720 | 120 min | -0.741 | 3.692 | 0.841 |

‡Mixed effects random intercept linear regression model, BMI or WC have no substantial effect on the outcomes. (**p < 0.01, *p < 0.05)
