# Supplementary material for: Phytosterols and inulin-enriched soymilk increases glucagon-like peptide-1 secretion in healthy men: double-blind randomized controlled trial, subgroup study
Source: BMC Res Notes. 2018 Nov 29;11:844. doi: 10.1186/s13104-018-3958-5 (PMC6267084; doi:10.1186/s13104-018-3958-5)
Supplement: Supplementary file 4 — Additional file 4. Mean differences of AUC of plasma glucose, insulin and GLP-1 between both groups (n = 25). [file 13104_2018_3958_MOESM4_ESM.docx]

**Additional file S4**. Mean differences of AUC of plasma glucose, insulin, and GLP-1 between the groups (n = 25).

^‡^ Mixed effects linear regression model

|  | Intervention (n = 14) | | | Placebo (n = 11) | | | p value^‡^ |
| --- | --- | --- | --- | --- | --- | --- | --- |
|  | Mean AUC (SD) | | Mean difference | Mean AUC (SD) | | Mean difference |  |
|  | Week 0 | Week 8 |  | Week 0 | Week 8 |  |  |
| glucose | 195.43 | 198.80 | 3.38 | 193.86 | 193.84 | -0.02 | 0.531 |
| (mg/dL) | (20.67) | (15.36) | (11.23) | (15.04) | (12.37) | (15.88) |  |
| Insulin | 38.84 | 44.38 | 5.54 | 47.98 | 43.93 | -4.04 | 0.073 |
| (mIU/L) | (30.10) | (24.69) | (8.98) | (20.65) | (18.30) | (17.31) |  |
| GLP-1 | 107.97 | 119.03 | 11.06 | 120.06 | 124.77 | 4.71 | 0.263 |
| (pg/mL) | (45.00) | (47.13) | (12.80) | (39.71) | (41.24) | (15.55) |  |
